# Association Between Chromogenic Black Stain and Dental Caries in Children: A Systematic Review and Meta-Analysis

**DOI:** 10.3390/children12121624

**Published:** 2025-11-30

**Authors:** Carla Borrell-Garcia, Paula Boo-Gordillo, Clara Guinot-Barona, Laura Marqués-Martínez, Esther Garcia-Miralles, Juan Ignacio Aura-Tormos

**Affiliations:** 1 Faculty of Medicine and Health Sciences, Catholic University of Valencia San Vicente Martir, 46001 Valencia, Spain; carla.borrell@ucv.es (C.B.-G.); clara.guinot@ucv.es (C.G.-B.); laura.marques@ucv.es (L.M.-M.); 2 Dentistry Department, Faculty of Medicine and Dentistry, University of Valencia, 46010 Valencia, Spain; m.esther.garcia@uv.es (E.G.-M.); juan.aura@uv.es (J.I.A.-T.)

**Keywords:** dental caries, black stain, children, meta-analysis

## Abstract

**Highlights:**

**What are the main findings?**

- Children with chromogenic black stain showed lower odds of dental caries compared with children without black stain in the studies included.
- The pooled prevalence of black stain in pediatric populations was approximately 9.4%, with considerable variability across studies.

**What are the implications of the main findings?**

- Current evidence suggests an inverse association between black stain and dental caries, although the heterogeneity across studies warrants cautious interpretation.
- Black stain is a benign extrinsic discoloration that may coincide with lower caries levels in some populations, but further research is required to clarify its potential relevance in caries risk assessment.

**Abstract:**

**Objectives**: To evaluate the association between chromogenic black stain and dental caries in children, and to estimate the pooled prevalence of BS in pediatric populations. **Methods**: A systematic search was conducted in PubMed, Web of Science, EMBASE, and the Cochrane Library up to February 2025. Eligible studies included observational studies, clinical trials, and systematic reviews on BS and dental caries in children ≤15 years. Data extraction and quality assessment (Newcastle–Ottawa Scale) were performed independently by two reviewers. Random-effects meta-analyses estimated pooled odds ratios for caries risk and BS prevalence. **Results**: Fourteen studies met inclusion criteria. Twelve reported an inverse association between BS and caries, one showed no significant relationship, and one was inconclusive. Five studies were included in the meta-analysis, which indicated that children with BS had significantly lower odds of caries (pooled OR = 0.53; 95% CI: 0.36–0.78). The pooled prevalence of BS was 9.4% (95% CI: 6.6–12.2%), with substantial heterogeneity among studies. **Conclusions**: Black stain in children may be associated with a lower risk of dental caries, although this finding should be interpreted with caution given the limited number of studies and substantial heterogeneity.

## 1. Introduction

Dental caries is one of the most prevalent chronic diseases worldwide, affecting both children and adults [1]. It represents a major public health burden, with significant impacts on quality of life and substantial economic costs [2], particularly in pediatric populations.

Unlike dental caries, chromogenic black stain (BS) is a non-destructive dental condition that presents as an extrinsic discoloration characterized by dark dots or pigmented lines typically located along the cervical third of tooth crowns. It differs from other extrinsic stains such as those caused by tea, coffee, or tobacco [3,4,5]. Clinically, BS is harmless and reversible after prophylaxis but often causes esthetic concerns among patients and caregivers [6,7].

The prevalence of BS varies considerably, ranging from 2% to nearly 20% across different populations, with reports from Europe, Asia, and South America showing diverse estimates [8,9,10,11,12]. Interestingly, since the early 20th century, epidemiological studies have suggested that children with BS may present with a lower prevalence of dental caries, raising the hypothesis that BS could act as a marker of reduced cariogenic risk [5,9,13]. However, evidence has been inconsistent, with some studies confirming this inverse association while others reported no significant relationship [12,14]. Such heterogeneity likely reflects differences in study design, diagnostic criteria, population characteristics, and confounding factors.

Given its potential relevance for understanding caries susceptibility patterns, clarifying the relationship between BS and dental caries remains important. If confirmed, the presence of BS could contribute to a better understanding of caries susceptibility profiles in children.

A previous systematic review and meta-analysis by Mousa et al. (2022) [9] provided initial evidence suggesting a protective association between BS and dental caries. However, that review was constrained by a limited evidence base and called for further high-quality studies. It did not include a pooled estimate of BS prevalence—an important epidemiological measure for clinicians and public health planning—and was restricted mainly to specific geographical regions. Moreover, its assessment of methodological quality was less comprehensive, which may have limited the generalizability of its conclusions.

The present systematic review and meta-analysis was designed to address these limitations by incorporating the most recent evidence, including studies published up to 2025; performing, for the first time, a meta-analysis to estimate the pooled prevalence of BS in pediatric populations; encompassing a broader range of study designs and geographical regions to enhance generalizability; and applying a rigorous and transparent risk-of-bias assessment to strengthen the methodological appraisal of the evidence.

We hypothesized that the presence of BS is associated with a lower risk of dental caries in children.

## 2. Materials and Methods

### 2.1. Protocol and Registration

This systematic review and meta-analysis were conducted in accordance with the PRISMA guidelines, and the review protocol was prospectively registered in PROSPERO (CRD420251154941).

### 2.2. Research Question

The research question was formulated according to the PECO framework, which is appropriate for observational studies. The Population (P) comprised children aged 0–15 years with primary, mixed, or permanent dentitions. The Exposure (E) was the presence of chromogenic black stain, while the Comparison (C) referred to children without black stain. The Outcome (O) was the presence or experience of dental caries.

### 2.3. Eligibility Criteria

The inclusion criteria encompassed observational studies (cross-sectional, cohort, and case–control), and clinical trials that evaluated the relationship between black stain and dental caries in children aged 0–15 years. Exclusion criteria comprised case reports, expert opinions, and secondary research (systematic reviews, meta-analyses, or umbrella reviews), as well as studies conducted in adult populations or in children receiving antibiotic or iron supplementation.

Included studies were categorized according to whether they assessed the association between black stain and dental caries or reported the prevalence of black stain. This classification was used to structure the quantitative synthesis.

The age range of 0–15 years was defined to include all stages of pediatric dentition—primary, mixed, and early permanent—where chromogenic black stain is most frequently reported. This range also covers the period of initial oral microbiota establishment, during which vertical bacterial transmission from mother to child may occur, allowing a comprehensive assessment of BS occurrence and its association with dental caries throughout childhood.

### 2.4. Information Sources and Search Strategy

A comprehensive search was conducted in PubMed, Web of Science, EMBASE, and the Cochrane Library up to February 2025, using the terms “black stain tooth” AND “caries” AND “children”. The search was restricted to studies published in English. The search was limited to these databases, and no additional sources or grey literature were explored. The full Boolean search strategy for all databases (PubMed, Web of Science, EMBASE, and the Cochrane Library) has been provided in the Supplementary Materials to ensure transparency and reproducibility.

### 2.5. Study Selection and Data Extraction

Two reviewers independently screened titles, abstracts, and full texts, and extracted data from the included studies. Discrepancies were resolved through discussion and mutual consensus, without the need for a third reviewer. The level of inter-reviewer agreement was calculated using Cohen’s kappa coefficient, which showed a substantial level of agreement (κ = 0.82).

Extracted data included study identification details (authors, year, and country), study design, sample size, participants’ age range, type of dentition examined, diagnostic criteria for black stain and dental caries, prevalence data, measures of association (e.g., odds ratios or relative risks), and information on confounding variables or microbiological analyses when available. When effect estimates were not directly reported, they were calculated or approximated from available data (e.g., event counts or mean values). Any discrepancies or uncertainties were resolved through discussion and consensus between reviewers.

For cohort studies, prevalence data were extracted when available. No restriction was applied regarding the duration of follow-up, provided that outcome assessment was clearly reported.

### 2.6. Risk of Bias Assessment

Study quality was appraised using two complementary approaches. The Oxford Centre for Evidence-Based Medicine (OCEBM) levels of evidence were applied to provide an overall classification of methodological strength for each study design. In parallel, the Newcastle–Ottawa Scale (NOS) was used to assess the risk of bias in individual observational studies across the domains of selection, comparability, and outcome or exposure assessment.

To facilitate interpretation, NOS scores were translated into qualitative categories following established thresholds: 7–9 stars = Low risk of bias, 4–6 stars = Moderate risk, and 0–3 stars = High risk. The qualitative categories used in the assessment correspond to the four domains of NOS: Selection, Comparability, Outcome/Exposure Assessment, and the Overall rating.

### 2.7. Statistical Analysis

Random-effects meta-analyses were performed. For association studies, pooled odds ratios (ORs) with 95% confidence intervals (CI) were calculated. For prevalence, pooled estimates were calculated using proportions with 95% CI. Heterogeneity was assessed with the I^2^ statistic.

Publication bias was planned to be evaluated through visual inspection of funnel plots and, when applicable, Egger’s regression test. However, this analysis was to be conducted only when at least ten studies were available per outcome, following current Cochrane and PRISMA recommendations.

For the prevalence meta-analysis, raw proportions were pooled without transformation, as none of the included estimates approached boundary values (0% or 100%) that would warrant logit or double–arcsine transformation.

## 3. Results

**Figure 1:**
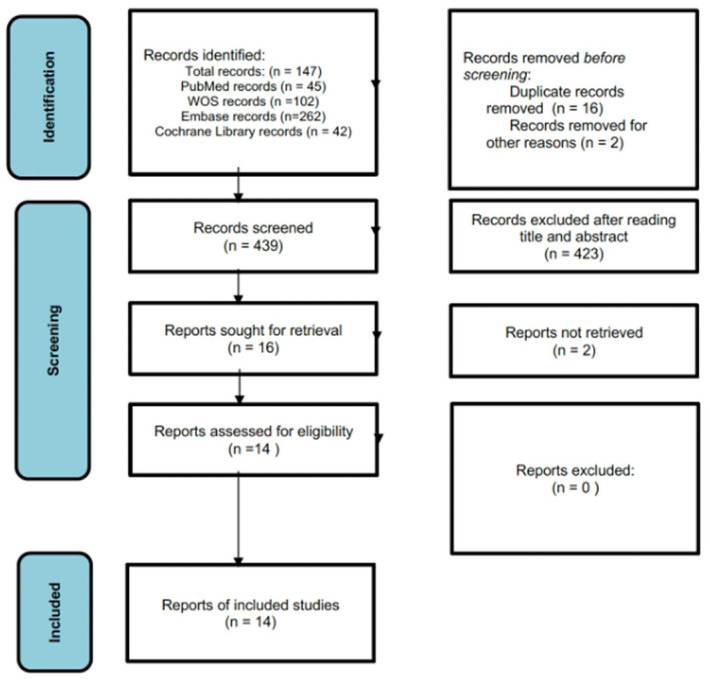
PRISMA flow diagram of study selection.

A total of 451 records were retrieved from the database search (45 from PubMed, 102 from Web of Science, 262 from EMBASE, and 42 from the Cochrane Library). After removing 12 duplicates, 439 records remained for screening. Following title and abstract review, 425 non-relevant records were excluded. Four reports were automatically marked as ineligible and two were removed for other reasons. Fourteen full-text articles were assessed for eligibility, and all met the inclusion criteria. No additional studies were identified through manual searching. A detailed description of the selection process is presented in Figure 1.

### 3.1. Study Characteristics

The 14 included studies were published between 2001 and 2024 and conducted across Europe, South America, Asia, and Africa. Eight were cross-sectional, four cohort, and two case–control designs, with sample sizes ranging from 38 to 2777 participants. Most studies evaluated primary dentition, while several also included mixed or permanent dentitions. Diagnostic criteria for black stain and dental caries varied across studies, contributing to methodological heterogeneity. Reported prevalence of black stain ranged from 2% to 18%.

Key characteristics of the included studies are summarized in Table 1.

### 3.2. Risk of Bias in Included Studies

The methodological quality and risk of bias were appraised using NOS. As shown in Table 2, five studies were rated as Low risk of bias (7–9 stars), four as Moderate (4–6 stars), and five as High (0–3 stars). Most studies adequately described participant selection and outcome assessment but showed variability in comparability of groups and control for confounding factors. No study met all NOS criteria across domains.

### 3.3. Quantitative Synthesis

A quantitative synthesis was conducted for studies providing sufficient data for effect size estimation. For inclusion in the meta-analysis, studies were required to report, or allow calculation of, the number of participants with and without black stain according to caries status (for association analyses) or the total number of participants and cases of black stain (for prevalence analyses).

#### 3.3.1. Association Between Black Stain and Dental Caries

**Figure 2:**
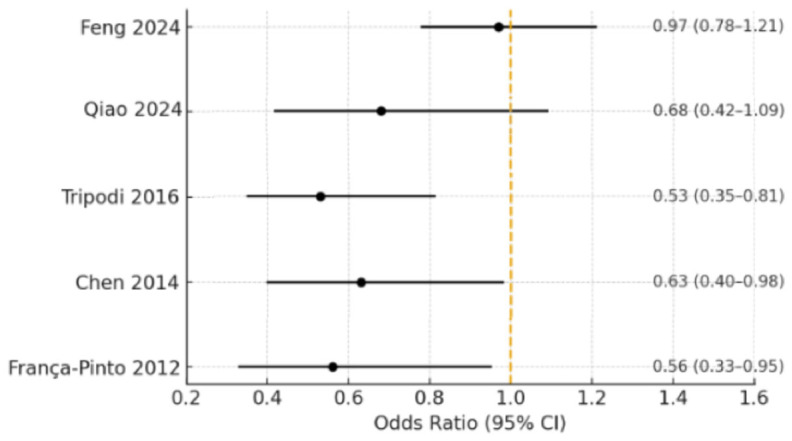
Forest plot of the association between black stain and dental caries *. * Subgroup analyses by dentition type (primary, mixed, or permanent) were considered; however, available data from the included studies were insufficiently detailed to support separate quantitative synthesis. Therefore, pooled estimates are presented for the overall pediatric population [5,6,7,8,18].

Five studies—França-Pinto et al. (2012) [7], Chen et al. (2014) [8], Tripodi et al. (2016) [18], Feng et al. (2024) [6], and Qiao et al. (2024) [5]—provided sufficient outcome data for quantitative synthesis. The pooled analysis indicated that the presence of BS was associated with lower odds of dental caries, although this estimate is based on only five studies and should be interpreted cautiously (OR = 0.53; 95% CI: 0.36–0.78; *p* < 0.01). Moderate heterogeneity was observed (I^2^ = 52%). The corresponding forest plot is shown in Figure 2.

#### 3.3.2. Prevalence of Black Stain

**Figure 3:**
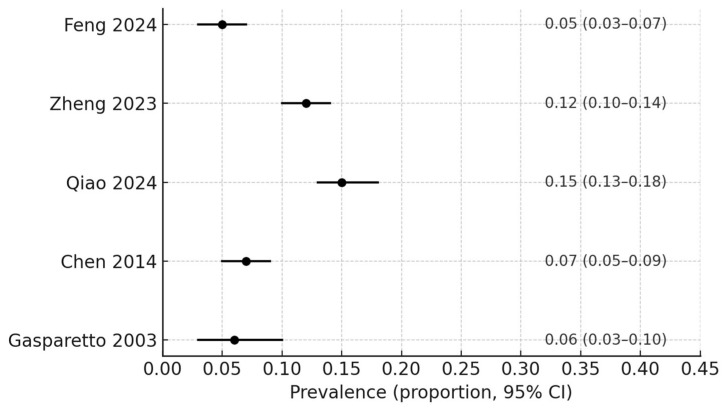
Forest plot of pooled prevalence of black stain in pediatric populations *. * Subgroup analyses by dentition type (primary, mixed, or permanent) were considered; however, available data from the included studies were insufficiently detailed to support separate quantitative synthesis. Therefore, pooled estimates are presented for the overall pediatric population [5,6,8,12,13].

Five studies—Gasparetto et al. (2003) [12], Chen et al. (2014) [8], López-Martínez et al. (2016) [11], Elelmi et al. (2020) [19], and Qiao et al. (2024) [5]—reported data suitable for pooling prevalence estimates. The overall pooled prevalence of BS was 9.4% (95% CI: 6.6–12.2%), with substantial heterogeneity across studies (I^2^ = 74%). The corresponding forest plot is presented in Figure 3.

#### 3.3.3. Publication Bias

The assessment of publication bias was not performed, as the limited number of studies precluded a meaningful analysis. This approach is consistent with current methodological recommendations, which advise against formal evaluation of publication bias in analyses including fewer than ten studies.

## 4. Discussion

This systematic review and meta-analysis suggest a possible inverse relationship, but the limited number of studies included in the quantitative synthesis reduces the strength of this evidence. The overall body of evidence shows a consistent trend across diverse populations and study designs, supporting the hypothesis that BS may coincide with lower caries susceptibility.

Our results are consistent with several epidemiological investigations. França-Pinto et al. (2012) [7] reported significantly lower caries prevalence among Brazilian preschoolers with BS. In China, Qiao et al. (2024) [5] similarly demonstrated reduced caries experience in children with BS. In Europe and Asia, Koch et al. (2001) [15] and Heinrich-Weltzien et al. (2009) [10] found that Italian and Filipino children, respectively, showed lower caries prevalence than their peers without BS. A previous systematic review and meta-analysis by Mousa et al. (2022) [9] reached comparable conclusions, providing supportive background evidence but was not included in the present quantitative synthesis as it was a secondary analysis.

However, other studies reported mixed outcomes. Elelmi et al. (2020) observed an association between BS and reduced early childhood caries but found no significant differences in mean dmft scores [19]. Similarly, Velásquez Sáez et al. (2017) reported no significant association between BS and salivary *Streptococcus mutans* counts, suggesting that not all microbial risk factors are mitigated by the presence of BS [14]. These discrepancies highlight the influence of study design, diagnostic thresholds, and population.

Several mechanisms have been proposed to explain the protective association of BS against caries. As described in earlier epidemiological studies, black stain has been associated with higher proportions of *Actinomyces* spp. and pigmented anaerobes such as *Prevotella* spp., as well as the presence of iron-containing compounds including ferric sulphide. These microbiological characteristics have been hypothesized to contribute to a less acidogenic biofilm environment, which could help explain the lower caries levels observed in some populations. Microbiological analyses reveal that the BS biofilm is compositionally distinct from conventional cariogenic plaque. It tends to be enriched in *Actinomyces* spp. and *Prevotella* species, organisms considered less acidogenic than *Streptococcus mutans*, the principal cariogenic bacterium [3,17,18,20]. Furthermore, salivary analyses indicate that children with BS often exhibit higher buffering capacity, increased calcium concentration, and more favorable pH values, all of which contribute to an oral environment less conducive to demineralization [16]. The deposition of ferric sulfide particles within BS may also help stabilize plaque pH, reducing acidogenic challenges to the enamel. Collectively, these factors may explain why BS is associated with reduced caries risk. However, these explanations are supported only by a small number of observational studies, and no mechanistic causal pathway has been demonstrated experimentally. Therefore, these hypotheses should be interpreted as exploratory rather than confirmatory.

This study has several strengths, including a comprehensive literature search across major databases, adherence to PRISMA guidelines, and the use of robust meta-analytic methods. Moreover, included studies represented diverse geographic regions, enhancing the generalizability of findings.

Nonetheless, several limitations should be acknowledged. First, only a small subset of the eligible studies provided sufficient data for meta-analysis, which substantially reduces statistical power. Second, the heterogeneity across studies was considerable—in terms of diagnostic criteria, population characteristics, and analytical approaches—further limiting the comparability and strength of the pooled estimates. Most of the included studies were observational in design, and therefore subject to potential risks of bias and residual confounding. Furthermore, considerable variability was observed in both prevalence and association estimates, reflecting differences in diagnostic criteria for BS and caries, study populations, and adjustment for potential confounders such as diet, oral hygiene, and fluoride exposure.

In exploratory terms, the moderately high heterogeneity observed in the association meta-analysis (I^2^ = 74%) may reflect differences among studies in age distribution, dentition type (primary vs. mixed), and geographical context. These factors vary substantially across the included studies and likely contributed to the variability in effect estimates. As the number of pooled studies was small, no formal subgroup analyses were feasible, and this interpretation should be considered descriptive rather than confirmatory.

In addition, publication bias cannot be ruled out, as studies with null results may be underrepresented. Language and selection biases are also possible, since only English-language publications were considered and grey literature or unpublished data were not searched. Although this restriction helps maintain methodological consistency, it may have resulted in the omission of relevant studies.

Finally, the literature search was limited to four major databases (PubMed, Web of Science, EMBASE, and the Cochrane Library), which were selected for their extensive biomedical coverage and indexing quality. The decision not to extend the search to additional databases or grey literature aimed to maintain a focused and reproducible strategy; however, this may have resulted in the omission of some relevant studies and could have affected the overall comprehensiveness of the review.

From a clinical perspective, BS should not be interpreted as having any direct clinical application based on current evidence; rather, it represents an observational finding that requires further investigation.

Importantly, many of the included studies were rated as having a high risk of bias, particularly regarding selection procedures, comparability of groups, and outcome assessment. This substantially reduces the confidence in the observed associations and limits the certainty and generalizability of the pooled estimates.

Future research should prioritize longitudinal cohort studies with standardized diagnostic criteria to confirm the temporal relationship between BS and caries development. Microbiome-based investigations are also warranted to further elucidate the ecological and biochemical mechanisms underlying the apparent protective effect.

Establishing a clear biological basis could facilitate the interpretation of these findings. BS may coincide with lower caries levels in some populations; however, the limited statistical power of the available evidence prevents drawing firm conclusions, and the methodological weaknesses of several studies likely influenced both the magnitude and direction of the estimates.

## 5. Conclusions

This systematic review and meta-analysis indicate a possible inverse association between black stain and dental caries, but this finding is based on a limited number of studies and considerable heterogeneity across populations and methodologies. Therefore, no clinical implications should be inferred from these results, and further well-designed longitudinal studies are needed to clarify the nature and consistency of this association and to better understand the potential mechanisms linking black stain to caries risk.

## Figures and Tables

**Table 1:** Main characteristics of included studies.

Author (Year)	Country	Study Design	Sample Size	Dentition Type	Prevalence of Black Stain (%)	Caries Outcomes	Main Findings
**Koch et al. (2001)** [15]	Italy	Cohort	1086	Mixed, permanent	6.3	DMFT	Significantly lower caries prevalence in children with BS
**Gasparetto et al. (2003)** [12]	Brazil	Cohort	263	Mixed, permanent	14.8	dmft/DMFT	Lower caries indices in children with BS
**Heinrich-Weltzien R et al. (2009)** [10]	Philippines	Cross-sectional	1748	Mixed	–	DMFT	Caries prevalence: BS 59%/No BS 81%
**França Pinto et al. (2012)** [7]	Brazil	Cohort	1120	Primary	3.5	Caries prevalence 48.4%	Lower caries prevalence in BS group
**Garan et al. (2012)** [16]	Turkey	Cohort	38	Mixed	–	dmft	Negative correlation between BS and dmft (*p* = 0.037)
**Weltzien et al. (2013)** [17]	Germany	Case–control	93	Primary, mixed, permanent	–	dmft/DMFT	Lower caries prevalence in primary dentition with BS
**Chen et al. (2014)** [8]	China	Cross-sectional	1397	Primary	9.9	dmft	Caries prevalence: BS 46.4%/No BS 59.1%
**Tripodi et al. (2016)** [18]	Italy	Case–control	189	Primary, mixed	–	dmft/DMFT	Lower active caries prevalence in children with BS
**López Martínez et al. (2016)** [11]	Brazil	Cross-sectional	1211	Mixed, permanent	5.8	DMFT	Lower mean caries scores in children with BS
**Velásquez-Sáez C et al. (2017)** [14]	Chile	Cross-sectional	158	Mixed (primary + permanent)	9.5	ceo-d/COP-D/*S. mutans* count	No significant difference *S. mutans* counts BS(+) and BS(−); BS not assoc. w/caries risk lower caries risk
**Elelmi et al. (2020)** [19]	Tunisia	Cross-sectional	393	Primary	6.1	dmft	BS associated with reduced ECC prevalence
**Zheng et al. (2023)** [13]	China	Case–control	2331	Primary	12.1	dmft	Lower prevalence of caries in BS group (*p* < 0.01)
**Feng et al. (2024)** [6]	China	Cross-sectional	2777	Primary, mixed, permanent	4.7	dmft/DMFT	OR = 0.23 (protective association)
**Qiao et al. (2024)** [5]	China	Cross-sectional	672	Primary	15.3	Caries prevalence 55.6%	Children BS (+) lower risk of caries (*p* < 0.001)

**Table 2:** Risk of bias assessment of the included studies according to the Newcastle–Ottawa Scale *.

Author (Year)	Risk of Bias (Study Type)	Selection	Comparability	Outcome/Exposure Assessment	Overall
**Koch MJ et al. (2001)** [15]	Low	Low	Moderate	Low	Low
**Gasparetto A et al. (2003)** [12]	Low	Low	Moderate	Low	Low
**Heinrich-Weltzien R et al. (2009)** [10]	High	High	High	Moderate	High
**Garan A et al. (2012)** [16]	Low	Low	Moderate	Low	Low
**França-Pinto CC et al. (2012)** [7]	Low	Low	Moderate	Low	Low
**Heinrich-Weltzien R et al. (2013)** [17]	Moderate	Moderate	Moderate	Moderate	Moderate
**Chen X et al. (2014)** [8]	High	High	High	Moderate	High
**López-Martínez TM et al. (2016)** [11]	High	High	High	Moderate	High
**Tripodi D et al. (2016)** [18]	Moderate	Moderate	Moderate	Moderate	Moderate
**Velásquez-Sáez C et al. (2017)** [14]	High	High	High	Moderate	High
**Elelmi Y et al. (2020)** [19]	High	High	High	Moderate	High
**Zheng L et al. (2023)** [13]	Moderate	Moderate	Moderate	Moderate	Moderate
**Feng J et al. (2024)** [6]	High	High	High	Moderate	High
**Qiao C et al. (2024)** [5]	High	High	High	Moderate	High

* The overall risk of bias was classified as: Low (7–9 stars), Moderate (4–6 stars), or High (0–3 stars). The assessment for each domain (Selection, Comparability, Outcome/Exposure) is also presented.

## Data Availability

The datasets generated and analyzed during the current study are available from the corresponding author.

## Supplementary Materials

The following supporting information can be downloaded at: https://www.mdpi.com/article/10.3390/children12121624/s1, Supplementary Annex S1.

## Abbreviations

BS	Black stain
DMFT	Decayed, Missing, and Filled Teeth (permanent dentition)
dmft	Decayed, Missing, and Filled Teeth (primary dentition)
ECC	Early Childhood Caries
OCEBM	Oxford Centre for Evidence-Based Medicine
NOS	Newcastle–Ottawa Scale
PRISMA	Preferred Reporting Items for Systematic Reviews and Meta-Analyses
PROSPERO	International Prospective Register of Systematic Reviews
OR	Odds Ratio
CI	Confidence Interval
I^2^	I-squared (measure of heterogeneity)
PECO	Population, Exposure, Comparison, Outcome
WHO	World Health Organization
*S. mutans*	*Streptococcus mutans*
pH	Hydrogen potential
SD	Standard Deviation (*implied though not explicitly shown*)
n	Sample size (used in data tables)
