# Supplementary material for: Association Between Chromogenic Black Stain and Dental Caries in Children: A Systematic Review and Meta-Analysis
Source: Children (Basel). 2025 Nov 30;12(12):1624. doi: 10.3390/children12121624 (PMC12731960; doi:10.3390/children12121624)
Supplement: Supplementary file 1 [file children-12-01624-s001.zip › children-3994945-supplementary.pdf]

SUPPLEMEMENTARY ANNEX 1

PubMed (MEDLINE)

“black stain” OR “black tooth stain” OR “extrinsic black stain”
OR (“black” AND “stain” AND “tooth”)
AND
“dental caries” OR caries OR dmft OR DMFT
AND
child OR children OR pediatric OR paediatric

Filters applied: English, Humans, Child (0–15 years).

Web of Science (Core Collection)

(“black stain” OR “black tooth stain” OR “extrinsic black stain”)
AND
(“dental caries” OR caries OR dmft OR DMFT)
AND
(child* OR pediatric* OR paediatric*)

Document types: Article, Review.
Language: English.

EMBASE (Elsevier)

“black stain” OR “black tooth stain” OR “extrinsic black stain”
AND
“dental caries” OR caries OR dmft OR DMFT
AND
child OR children OR pediatric OR paediatric

Limits applied: Humans, English language, Child (0–15 years).

Cochrane Library

“black stain” OR “black tooth stain” OR “extrinsic black stain”
AND
“dental caries” OR caries OR dmft OR DMFT

AND
child OR children OR pediatric OR paediatric

Sources searched: Trials, Reviews, CENTRAL.
